# False-positive rapid diagnostic tests in paediatric and obstetric patients in South Africa

**DOI:** 10.4102/sajhivmed.v22i1.1186

**Published:** 2021-01-29

**Authors:** Josephine Keal, Ahmad H. Mazanderani, Nicola van Dongen, Gillian Sorour, Karl-Gunter Technau

**Affiliations:** 1 Empilweni Services and Research Unit, Rahima Moosa Mother and Child Hospital, Department of Paediatrics and Child Health, Faculty of Health Sciences, University of the Witwatersrand, Johannesburg, South Africa; 2 Centre for HIV and STIs, National Institute for Communicable Diseases, National Health Laboratory Service, Johannesburg, South Africa

**Keywords:** HIV, rapid diagnostic test, discordant, diagnostic dilemmas, pregnancy

## Abstract

**Introduction:**

Providing easily accessible, quick and accurate human immunodeficiency virus (HIV) testing services (HTS) is central to achieving the Joint United Nations Programme on HIV/AIDS (UNAIDS) 90-90-90 targets. Rapid diagnostic tests (RDTs) for HIV are affordable and technically easy to perform. Two positive RDTs from different manufacturers are required to make a diagnosis of HIV in South Africa. Difficulty arises when there are discordant results from the two kits. In this case report, we will discuss four instances of false-positive RDTs.

**Patient presentation:**

Case 1 is a 10-year-old female, referred for initiation of antiretroviral treatment (ART). She was diagnosed using two of the same brand RDT at her local clinic. Case 2 is a 21-year-old female who presented to obstetric admissions in labour. Case 3 is a 39-year-old female who was screened for HIV during a routine antenatal appointment. Case 4 is a 22-year-old female who was admitted 21 days postpartum with puerperal sepsis. All four cases had discordant RDTs when screened for HIV at our facility.

**Management and outcome:**

The results of all the investigations conducted on all four patients confirmed HIV negative status. The reference laboratory verified the results and reran the RDTs, which remained discordant. This confirmed a false-positive result in all four cases with the screening RDT.

**Conclusion:**

With high numbers tested and a low yield of new cases, each individual case of discordancy may cause unnecessary distress, confusion and treatment, particularly in high-risk scenarios like pregnancy. Trends of false-positive and discordant RDT results should be monitored and inform HTS guidelines.

## Introduction

Accessible, quick and accurate human immunodeficiency virus (HIV) testing services (HTS) are imperative to achieving the Joint United Nations Programme on HIV/AIDS (UNAIDS) 90-90-90 targets.^1^ Paediatric and obstetric HTS are particularly important entry points into care. Correct diagnosis facilitates important decisions around maternal antiretroviral therapy (ART), breastfeeding choices and infant prophylaxis. South African guidelines recommend ART initiation following two consecutive HIV-detected rapid diagnostic tests (RDTs), using assays from different manufacturers, at a single time-point.^2^

Our centre uses two World Health Organization (WHO)-approved RDT kits for HIV diagnosis in all individuals older than 2 years.^3^ Abon HIV 1/2/0 (Abon Biopharm, Hangzhou, China) is used as the screening test (ScreenRDT). In the case of a positive result, a First Response HIV 1–2.0 Card test (Premier Medical Corporation Private, Ltd., Gujarat, India) is done as a confirmation test (ConfirmRDT). Screening rapid diagnostic test has a reported specificity of 99.7%, ConfirmRDT has a specificity of 100%.^3^ As the HIV prevalence is decreasing in the tested population and the ScreenRDTs we use do not have 100% specificity, an increasing proportion of false-positive RDT results can be expected. This is a result of the decreasing positive-predictive value of the tests used, as they are dependent on test specificity and disease prevalence. Human immunodeficiency virus testing yield in South African adults has dropped over the past decade to ~5%^4^; thus, the expected false-positivity rate may reach 5%.^5^ If the national HTS guidelines are not followed correctly, false positives may cause individuals to be erroneously initiated on ART.^6^

**FIGURE 1:**
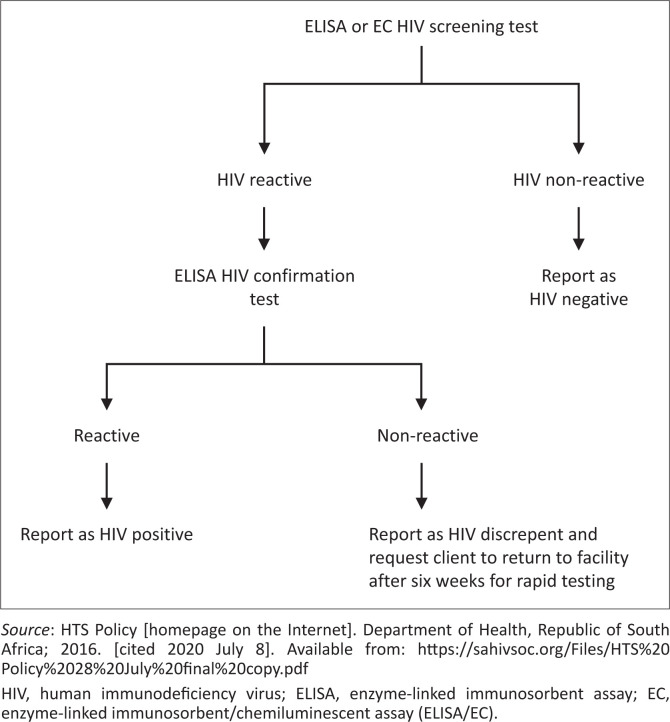
Reflex human immunodeficiency virus laboratory testing to resolve discrepant human immunodeficiency virus rapid testing.^2^

Complications can arise when ScreenRDT and ConfirmRDT are discordant, with one test indicating a reactive result and the other test a non-reactive result. If the discordant result is still present after the RDT algorithm is repeated, further tests are warranted. As per the national HTS guidelines the tiebreaker test is a 4th-generation HIV enzyme-linked immunosorbent assay (ELISA), such as the Architect HIV-1/2 Ag/Ab combo assay (Abbott Laboratories, Wiesbaden, Germany) used in this case report (Figure 1).^2^ If the ELISA yields equivocal results, a qualitative HIV polymerase chain reaction (PCR) test [COBAS AmpliPrep/TaqMan HIV-1, v2.0; Roche Molecular Systems, Branchburg, New Jersey, United States of America (USA)] is recommended.

In this case report, we discuss four instances of likely false-positive ScreenRDT to sensitise clinicians to the importance of following the HTS algorithm correctly and to the unique situations, dilemmas and potential risks faced in high-risk circumstances.

The tiebreaker tests are performed in central laboratories and have an expected turnaround time (TAT) of less than 3 days for the HIV ELISA and less than 1 week for the HIV PCR. Our facility is located in an urban area and is affiliated with a university; therefore, the TAT on the samples was significantly quicker than the provincial average (Table 1).

**TABLE 1:** Turnaround times for tests done and final results.

Point-of-care tests				Confirmatory tests			Time to confirmed negative result (RDT + ELISA TAT)
	RDT†	POC PCR	POC VL	VL	ELISA	Western blot	
	
Results	Discordant	Negative	LDL	LDL	Negative	Negative	
	
TAT	Minutes			Hours			
Case 1	-	-	-	18	8	51	9
Case 2	60	90	90	40	12	113	13
Case 3	-	-	-	59	4	49	5
Case 4	-	-	-	86	15	46	16
Mean	60	90	90	51	10	65	11

POC, point of care; PCR, polymerase chain reaction; VL, viral load; ELISA, enzyme-linked immunosorbent assay; RDT, rapid diagnostic test; TAT, turnaround time; LDL, Lower than detectable limit.

† , Time includes running the screening and confirmation RDTs, as well as repeating the testing algorithm.

All four cases discussed presented with discordant RDTs – a positive ScreenRDT and negative ConfirmRDT. Because of the high-risk and time-sensitive nature of the cases, point-of-care tests available on site at the time [Cepheid Xpert^TM^ HIV-1 VL (viral load) and Xpert HIV-1 PCR, Sunnyvale, California, USA] as well as tests indicated by the national testing algorithm were conducted. In addition, a Western blot assay was done on all four cases after discussion with the reference laboratory.

Case 1 was a healthy 10-year-old female with no previous admissions or chronic conditions. Her mother had been living with HIV since before Client 1 was born; thus our patient was HIV exposed at birth. She had been tested three times at her local clinic according to her mother, with negative results (which we could not verify). Her mother defaulted treatment when Client 1 was 7 years old. Efforts were made by the local clinic to invite the mother back into care. She returned and was asked to bring her children in for testing. Client 1 was brought in for index testing at the time. She was diagnosed as HIV infected using two rapid tests (two ScreenRDT kits were used at the clinic, because of stock-out of the confirmatory kit). She was referred to our facility for treatment initiation as they did not have stock of the appropriate ART. At our facility, she was found to be asymptomatic, and no history or suspicion of sexual abuse was reported. Despite the referral letter requesting ART initiation only, the rapid tests were repeated, yielding a ScreenRDT positive and ConfirmRDT negative results. The RDTs were repeated, with discordant results again.

Our second case was a 21-year-old female who presented to obstetric admissions in labour at 39 weeks pregnant. As per national HTS guidelines she was screened for HIV on admission. Her ScreenRDT result was positive and ConfirmRDT negative. The patient had been screened at every antenatal visit (four times) prior to delivery. All prior screenRDT results were negative. She was counselled on her discordant results and the risks associated with HIV and labour and agreed to take stat doses of tenofovir-lamivudine-dolutegravir (TLD) fixed-dose combination and nevirapine whilst awaiting the confirmatory tests.

Case 3 was a 39-year-old female in her third trimester of pregnancy. She was screened for HIV during a routine antenatal care (ANC) visit, with discordant results. She had tested negative at her ANC visit a month previously. She conveyed a high level of anxiety about the positive result as she, reportedly, had not been involved in any high-risk behaviour since the conception of her child. Her partner had been out of the country for the past 5 months.

Case 4 was a 22-year-old female admitted 21 days postpartum with puerperal sepsis. The patient had tested negative four times during her pregnancy and at delivery. The positive result caused significant concern to the patient as she had been exclusively breastfeeding her child since birth.

The additional investigations conducted on all four patients showed HIV-negative results. The reference laboratory confirmed the results and reran the RDTs, which remained discordant. This confirmed a false-positive ScreenRDT result in all four cases.

## Discussion

Offering accurate, accessible HTS with a quick TAT is imperative to realising the UNAIDS 90-90-90 targets. With high numbers tested and a low testing yield of new cases, the positive predictive value of RDTs is dropping. Each individual case of discordancy is very important.

Discordant results create anxiety and uncertainty around a diagnosis for both the patient and the healthcare worker. This has the potential to delay treatment or cause doubt around a positive diagnosis.

False-positive results run the risk of patients being incorrectly initiated on treatment, as in our first case. Not only is this an unnecessary burden for the patient, but the expense of lifelong therapy or potential litigation could be significant for the state. In a study done in 2017 looking at misdiagnoses of HIV in pregnant women in South Africa,^6^ the cost per misdiagnosis was much larger than the cost of additional confirmatory testing.

Human immunodeficiency virus testing services and antenatal services in low- to middle-income countries still have multiple barriers to achieving effective service delivery.^7^ Diagnostic dilemmas in pregnancy add to the stress of delivery and pregnancy, both for the patient and the healthcare provider.

It is estimated that 35% – 40% of mother-to-child transmissions occur during labour and delivery.^8^ Women are particularly vulnerable to new HIV infection during pregnancy and the postpartum period, and recent infection may give rise to discordant RDT results.^9,10^ Diagnostic dilemmas during labour may delay administering ART in a high-risk situation. Further enquiries need to be made into the ScreenRDT by investigating factors that may falsely trigger the kit.^11^

The WHO strategy of using three positive RDTs to make a diagnosis of HIV could be considered, as it may avoid diagnostic dilemmas in pregnancy and other high-risk scenarios. It may possibly speed up the time to diagnosis, particularly in cases around delivery, where time is of the essence.^4^ In order to ensure appropriate management and counselling of these cases, all healthcare workers involved with HTS should be trained and sensitised to the risks around discordant results. Application of the South African guidelines would have sufficed in these cases. Reporting and monitoring of these cases must be emphasised in order to sensitise policymakers to the true number of discordant cases and implement protocols within facilities where there is a disproportionately high number.^12^
